# Process optimization of extrusion variables and its effect on properties of extruded cocoyam (*Xanthosoma sagittifolium*) noodles

**DOI:** 10.1002/fsn3.786

**Published:** 2018-10-02

**Authors:** Sunday Samuel Sobowale, Oluwatoyin Habibat Animashaun, Antoine Floribert Mulaba‐Bafubiandi, Temitope Saratallahi Abidoye, Yusuf Olamide Kewuyemi, Oluwafemi Ayodeji Adebo

**Affiliations:** ^1^ Department of Food Technology Moshood Abiola Polytechnic Abeokuta Ogun State Nigeria; ^2^ Department of Extraction Metallurgy Faculty of Engineering and the Built Environment University of Johannesburg Johannesburg Gauteng South Africa; ^3^ Department of Biotechnology and Food Technology Faculty of Science University of Johannesburg Johannesburg Gauteng South Africa

**Keywords:** Cocoyam, extrusion, noodles, optimization, quality attributes

## Abstract

The current industrial demand for starchy foods has been dominated by other roots and tubers, while cocoyam, despite being rich in fiber, minerals, and vitamins has remained under exploited. In this study, the effect of feed moisture content (FMC), screw speed (SS) and barrel temperature (BT) on the quality characteristics of cocoyam noodles (proximate, thermo‐physical, physicochemical, texture, color, extrudate properties, and sensory characteristics) were investigated using central composite design (CCD) of response surface methodology (RSM). Flour was produced from fresh tubers of cocoyam (*Xanthosoma sagittifolium*) and subsequently processed into noodles using a twin screw extruder. Results showed that the proximate compositions, thermo‐physical, physicochemical properties, and color of the cocoyam noodles were significantly (*p *< 0.05) influenced by the extrusion process variables. The texture and extrudate properties of cocoyam noodles were equally significantly (*p *< 0.05) different. The experimental data obtained and predicted values of the response models were comparable, with statistical indices [absolute average deviation (AAD, 0–0.23), bias factor (*B*_f_, 1–1.08), and accuracy factor (*A*_f_, 1–1.23)] indicating the validity of the derived models. The optimal extrusion processing conditions for quality cocoyam noodles were FMC, SS, and BT of 47.5%, 700 rpm and 50°C, respectively, as cocoyam noodles obtained at these conditions had comparable properties and were most preferred and accepted by the sensory panelists.

## 1 INTRODUCTION

Cocoyam (*Xanthosoma sagittifolium*) is an important staple food grown extensively in the Southern belt of Nigeria, and the country was the world's largest producer as at 2014 (FAOSTAT, 2016). The crop is preferred over other root and tuber crops as it has highly digestible starch and it is also rich in crude protein, calcium, phosphorus, pro‐vitamin A and B‐complex vitamins (Bown, 2000; Emmanuel‐Ikpemel, Eneji, & Essiet, 2007; Kaushal, Kumar, & Sharma, 2012). Due to its relatively high moisture content, cocoyam tubers are susceptible to deterioration after harvest. As such, cocoyam is further processed using different techniques to make it available in various forms including boiled, roasted, or fried forms and starch.

Food processing through extrusion technique involves numerous unit operations including mixing, kneading, shearing, cooking, shaping, and forming together for value addition to food commodities (Filli, Jideani, & Jideani, 2014; Sobowale, Ayodeji, & Adebiyi, 2017). Extrusion cooking is a high‐temperature, short‐time process in which food materials are cooked in a tube by a combination of moisture, pressure, temperature, and mechanical shear, resulting in molecular transformation, gelatinization, protein denaturation, and disruption of bonds leading to products with new shapes and textures (Castells, Marin, Sanchis, & Ramos, 2005; Gui, Gil, & Ryu, 2012; Sobowale, Ayodeji, & Adebiyi, 2017). Extrusion further leads to the reduction in anti‐nutritional factors, increase in product microbiological safety, and much better consumer acceptability (Korkerd, Wanlapa, Puttanlek, Uttapap, & Rungsardthong, 2016; Sumathy, Ushakumari, & Malleshi, 2007). This versatile processing technique has been applied to the development of various inexpensive food products such as instant beverages, snacks, pasta, baby foods, noodles, and remains potentially promising for the processing of cocoyam.

Knowledge of raw material properties including its thermo‐physical properties and the nature of its flow under set of conditions within the extruder is vital to control the extruder behavior for high quality end products (Changi, Martinez‐Bustos, & Larai, 1998; Yusuf, Filli, Umar, & Halilu, 2017). Thermo‐physical properties such as density, specific heat, thermal diffusivity, and thermal conductivity of the material are fundamentally important in the mathematical modeling studies for the design and optimization of food processing operation involving heat and mass transfer. Physicochemical properties on the other hand provide an effective method for characterizing changes in a food material during extrusion and may give accounts of thermal and mechanical effects and modifications thereof.

Despite its nutritional properties and huge production volumes in Africa, cocoyam is still under exploited, coupled with this is the dominance of other roots and tubers for meeting the current industrial demand for starchy foods (Emmanuel‐Ikpemel et al., 2007). Cocoyam nonetheless remains an indispensable crop that can potentially alleviate the current prevalent food insecurity in the continent. Extrusion is a viable food processing technology for the manufacture of relevant food products that could benefit both consumer and industries. In this context and to encourage the utilization of cocoyam for the production of value added foods, this study was aimed at investigating and evaluating the influence of extrusion variables (feed moisture content, screw speed, and barrel temperature) on the proximate, thermo‐physical, physicochemical, extrudate, color, texture, and sensory properties of cocoyam noodles.

## 2 MATERIALS AND METHODS

### 2.1 Processing of cocoyam to flour

Fresh cocoyam tubers (*Xanthosoma sagittifolium*) were purchased from Sagamu market (8.25°N 5.65°W), Ogun State, Nigeria. Cocoyam flour was subsequently produced using the method of Sobowale, Ayodeji, & Adebiyi (2017). The cocoyam tubers were washed with distilled water to removes and, dirt, and other adhering materials. The tubers were peeled, re‐washed and sliced (an average of 0.02 mm thickness) into distilled water containing food grade sodium metabisulfite (to prevent browning). The sliced cocoyam tubers were then placed in a sieve to remove excess water and dried in an oven (Gallemkamp Scientific, UK) at 50°C for 9 hr, milled, screened through a 0.25‐mm sieve and packaged in high‐density polyethylene bags prior to analysis.

### 2.2 Experimental design and process optimization

Response surface methodology (RSM) was used to build up a mathematical model and assisted in qualitatively interpreting and describing the relationships between the three independent extrusion variables feed moisture content (*X*_1_), screw speed rate (*X*_2_), and barrel temperature (*X*_3_). The three‐factor design gave a total of 20 experiments as presented in Table 1. The responses investigated in this study were color, texture, thermo‐physical, extrudate, and physiochemical properties. The regression model describing the relationship between the independent variables in terms of their linear, quadratic, and interaction effects is expressed by the second‐order empirical polynomial equation as presented in Equation (1).

**Table 1:** Coded and real values for the response surface methodology

Experimental runs	*X*_1_	*X*_2_	*X*_3_	FMC (%)	SS (RPM)	BT (°C)
1	1	1	1	52.5	800	65
2	−1	−1	0	42.5	600	55
3	1	−1	−1	52.5	600	45
4	1	−1	−1	42.5	800	45
5	0	1	−1	47.5	800	45
6	−1	1	−1	42.5	800	45
7	0	−1	1	47.5	600	65
8	0	0	0	47.5	700	55
9	0	−1	−1	47.5	600	45
10	1	−1	0	52.5	600	55
11	1	0	0	52.5	700	55
12	1	1	−1	52.5	800	45
13	0	0	−1	47.5	700	45
14	−1	1	0	42.5	800	55
15	0	1	0	47.5	800	55
16	0	0	1	47.5	700	65
17	0	−1	0	47.5	600	55
18	1	−1	1	52.5	600	65
19	−1	1	1	42.5	800	65
20	−1	0	0	42.5	700	55

*X*_1_: feed moisture content (FMC); *X*_2_: screw speed rate (SS); *X*_3_: barrel temperature (BT).

(1) $$\begin{array}{cc} Y= & \beta_{0}+\beta_{1}\text{FMC}+\beta_{2}\text{SS}+\beta_{3}\text{BT}+\beta_{11}{\text{FMC}}^{2}+\beta_{22}{\text{SS}}^{2}+\beta_{33}{\text{BT}}^{2}\\ & +\beta_{12}\text{FMC}*\text{SS}+\beta_{13}\text{FMC}*\text{BT}+\beta_{23}\text{SS}*\text{BT}+\varepsilon \end{array}$$

where *Y* is the predicted response, *β*_0_ is a constant, FMC is the feed moisture content, SS is the screw speed, BT barrel temperature, *β*_1_–*β*_3_, *β*_11_–*β*_33_ and *β*_12_–*β*_23_ are regression coefficients for intercept, linear, and quadratic effects, respectively.

### 2.3 Extrusion process of cocoyam noodles

The noodles were produced using the modified method of Sobukola, Babajide, and Ogunsade (2013) in a laboratory scale twin screw extruder. The fabricated extruder had a barrel diameter, nominal screw length, restriction die and power of 65.2 mm, 1898 mm, 2 mm, and 5 hp, respectively (Sobowale, Ayodeji, & Adebiyi, 2017). Dough was prepared by mixing 100% cocoyam flour with a predetermined amount of water, to bring the moisture level to the different desired experimental moisture contents (Table 1). The extruder was subsequently operated using the feed moisture content, screw speed, and barrel temperature combination obtained from the CCD experimental design (Table 1). After extrusion, the extrudates were cut into smaller pieces of 2.5 mm height each, cooled to 25°C, and packaged for subsequent analysis.

### 2.4 Proximate analysis

Proximate composition of noodles (moisture, protein, fat, crude fiber, ash, and carbohydrate) was determined using standard analytical methods of AOAC (2006).

### 2.5 Determination of thermo‐physical properties

#### 2.5.1 Density (*ρ*)

Five grams of each sample was weighed and put into 100 ml measuring cylinder containing 50 ml water (as floatation liquid) and the density determined using simple floatation principles (Sobowale, Awonorin, Shittu, & Ajisegiri, 2014). The density was derived from the mass of sample divided by volume occupied.

#### 2.5.2 Specific heat capacity (*C*_p_)

The specific heat capacity was determined using two lagged copper calorimeters (Hussain & Rahman, 1999; Sobowale et al., 2014). The specific heat capacity was determined as follows:

(2) $$C_{p}=1/M_{p}\left[M_{w}C_{w}G_{w}G_{p}-M_{c}C_{c}\right]/60$$

where *M*_p_, *M*_w_, and *M*_c_ are the mass of sample, water, and calorimeter, respectively; *C*_w_ and *C*_c_ are the specific heat capacity of water and calorimeter, respectively; *G*_w_ and *G*_p_ are the slope of cooling curve for water and sample, respectively.

#### 2.5.3 Thermal diffusivity (*α*)

The methods of Tong, Sheen, Shah, Huang, and Lund (1993) and Sobowale, Awonorin, et al. (2017) were adopted using a probe connected by *K*—thermocouple wires to an Alda AVD 890C^+^ digital multimeter. The temperature history of the sample was determined by inserting of the probe into the center (radial axis of the sample).

#### 2.5.4 Thermal conductivity (*K*_s_)

The thermal conductivity was estimated from the corresponding thermal diffusivity value and other thermo‐physical properties such as specific heat (*C*_p_) and bulk density (*ρ*) (Rapusas & Driscoll, 1995; Sobowale, Awonorin, et al. 2017). The thermal conductivity was then calculated using the expression in Equation (3).

(3) $$\alpha =K_{s}/\rho C_{p}\Rightarrow K_{s}=\alpha \rho C_{p}$$

where *K*_s_, *α*,* ρ*, and *C*_p_ are thermal conductivity, thermal diffusivity, bulk density, and specific heat capacity, respectively.

### 2.6 Determination of physicochemical properties

#### 2.6.1 Swelling capacity, solubility index, and water absorption capacity

Swelling capacity was determined using the method of Olatidoye and Sobowale (2011), while solubility index was determined using the method described by Singh, Raina, Bawas, and Saxena (2005). Water absorption capacity was determined using the method of Olatidoye and Sobowale (2011).

#### 2.6.2 Amylose and amylopectin content

The amylose and amylopectin contents of the cocoyam flour were determined using the iodine calorimetric method (Udachan, Sahoo, & Hend, 2012).

#### 2.6.3 Color analysis

Image acquisition was carried out using a color digital camera (Nikon Cool Pix l21, Nikon Corp., Tokyo, Japan) connected to a computer USB interface and mounted on a stand inside a large box impervious to light with black inner surfaces (Yam & Papadakis, 2004). The acquired images were stored in high resolution JPEG formats in RGB color coordinates. These were subsequently converted to CIELB or LAB values using Adobe Photoshop 6.0 software and normalized to L*, a*, b* values using Equations (4–6) (Yam & Papadakis, 2004).

(4) $$L^{*}=\frac{L}{255}\times 100$$

(5) $$a^{*}=\frac{a\times 240}{255}-120$$

(6) $$b^{*}=\frac{b\times 240}{255}-120$$

#### 2.6.4 Texture analysis (Hardness)

The hardness of the cocoyam noodle was determined using a texture analyzer (TA‐XT2i, stable micro system, Haslemere, UK) following the procedure of Da Silva and Moreira (2008), which consists of a three‐point bending test. Samples were placed on a metal support at a distance of 90 mm apart, and the force required to break the extrudates was determined by using a steel blade of 3 mm to snap the samples at a speed of 10 mm/s. The force (*N*) at the fracture point was used as the resistance to breakage.

### 2.7 Determination of extrudate properties

#### 2.7.1 Cooking time

Using the methods of Sanni, Bamgbose, and Sanni (2004), the cocoyam noodle was cooked by immersion in boiling water and thereafter allowed to stay for few minutes. The different time taken for each of the samples to cook was recorded.

#### 2.7.2 Expansion ratio

Expansion ratio (ER) was determined as described by Rosentrater, Muthukumarappan, and Kannadhason (2009) and Sobowale, Bamgbose, and Adeboye (2016). The diameter of each extrudate was measured with a Vernier caliper (STORM Index‐Temp model, Italy) and divided by the diameter of the die nozzle.

#### 2.7.3 Residence time

Residence time (RT) was determined during extrusion using the method of Iwe, Vanzuilichem, and Ngoddy (2001). A print of red food color was introduced at the feeding port, and the time taken for the color to first show up at the die orifice was recorded as the residence time.

#### 2.7.4 Mass flow rate

The mass flow rate (MFR) was determined when steady‐state operation conditions were reached as indicated by constant torque at the different extrusion temperatures (Oke, Awonorin, Sanni, Asiedu, & Aiyedun, 2013). A stopwatch was started immediately, and sample of extrudates flowing out of the extruder die opening was collected as soon as the timer was started at 60‐s interval. Mean weight of triplicate collections was calculated for each run.

### 2.8 Sensory evaluation of extruded cocoyam noodles

Prior to the sensory evaluation test, ethical clearance was obtained and informed consent of the sensory panelists were sort and gotten. Sensory evaluation of the extrudates was performed using 20 panelists. Each panelist was requested to assess each coded sample and to record the degree of difference using a 9‐point Hedonic scale, based on appearance, taste, chewiness, glossiness, firmness, and overall acceptability.

### 2.9 Statistical analysis

All analyses were carried out in triplicate and average of the triplicate determinations were represented in the results, expressed as mean and standard deviation. The data obtained were subjected to analysis of variance (ANOVA) using SPSS 22 software (IBM, USA). Significant *F* tests at *p* < 0.05 levels of probability are reported. Minitab 16 statistical software (Minitab Lt. Coventry, UK) was used in generating statistical models and also to execute ANOVA on the models at 5% confidence level. To validate the model equations obtained, the average absolute deviation (AAD), bias factor (*B*_f_), and accuracy factor (*A*_f_), were calculated using Equations (7), (8), (9). The coefficient of determination (*R* ^2^) was also generated to compare the experimental and predicted values given by the models.

(7) $$\text{AAD}=\frac{\left[\sum_{i=1}^{N}\left(\frac{Y_{i,\exp}-Y_{i,\mathrm{cal}}}{Y_{i,\exp}}\right)\right]}{N}$$

(8) $$B_{f}=10^{\frac{1}{N}}\sum_{i=1}^{N}\log\left(\frac{Y_{i,\mathrm{cal}}}{Y_{i,\exp}}\right)$$

(9) $$A_{f}=10^{\frac{1}{N}}\sum_{i=1}^{N}\left|\log\left(\frac{Y_{i,\mathrm{cal}}}{Y_{i,\exp}}\right)\right|$$

## 3 RESULTS AND DISCUSSION

### 3.1 Proximate composition

The moisture, ash content, fat content, fiber, protein, and carbohydrate content of the cocoyam noodles as presented in Table 2 ranged from 5.66%–12.26%, 0.47%–10.26%, 2.47%–8.98%, 0.18%–5.51%, 16.38%–33.46%, and 41.57%–66.65%, respectively. Noodles produced from FMC of 47.5%, SS of 800 rpm and BT of 55°C had the highest moisture content, while those obtained at same conditions but slightly lower SS of 700 rpm had the lowest. Though there were significance differences (*p* < 0.05) in the moisture content of the noodles, they were all ≤12%, indicative of a long shelf life when stored (Kure, Bahago, & Daneil, 1998; Olatidoye & Sobowale, 2011). There were significance differences in the ash content of the samples which was observed to be relatively high when the FMC was at 47.5%, BT 65°C and 700 rpm.

**Table 2:** Proximate composition of cocoyam noodles

*X*_1_ (%)	*X*_2_ (RPM)	*X*_3_ (°C)	MC (%)	Ash (%)	Fat (%)	Fiber (%)	Protein (%)	CHO (%)
52.5	800	65	8.45^e^ (0.01)	4.82^q^ (0.01)	4.85^i^ (0.01)	1.35^m^ (0.03)	25.94^h^ (0.08)	54.60^h^ (0.08)
42.5	600	55	8.96^g^ (0.01)	2.71^o^ (0.01)	8.58^n^ (0.04)	0.99^k^ (0.01)	26.23^i^ (0.30)	52.56^e^ (0.36)
52.5	600	45	9.67^k^ (0.01)	1.80^h^ (0.01)	5.78^k^ (0.02)	0.75^fg^ (0.04)	29.80^n^ (0.21)	52.21^d^ (0.17)
42.5	800	45	8.52^f^ (0.01)	4.47^p^ (0.01)	8.65^o^ (0.01)	1.26^l^ (0.04)	23.68^g^ (0.02)	53.44^f^ (0.02)
47.5	800	45	10.92^n^ (0.01)	1.93^j^ (0.02)	4.06^e^ (0.01)	0.67^e^ (0.01)	26.62^j^ (0.01)	55.82^j^ (0.00)
42.5	800	45	7.95^d^ (0.03)	1.23^c^ (0.02)	6.52^l^ (0.02)	0.32^c^ (0.02)	31.67^p^ (0.01)	52.33^d^ (0.92)
47.5	600	65	9.96^m^ (0.04)	1.86^i^ (0.01)	3.67^d^ (0.03)	1.00^k^ (0.01)	33.46^q^ (0.04)	50.07^c^ (0.02)
47.5	700	55	5.66^a^ (0.08)	9.30^s^ (0.01)	3.43^c^ (0.02)	4.64^o^ (0.04)	18.18^b^ (0.06)	58.81^m^ (0.05)
47.5	600	45	9.30^j^ (0.02)	2.03^k^ (0.02)	4.95^j^ (0.04)	0.71^ef^ (0.01)	16.38^a^ (0.01)	66.65^s^ (0.03)
52.5	600	55	10.88^n^ (0.01)	2.10^l^ (0.01)	4.51^g^ (0.01)	0.74^fg^ (0.02)	18.51^c^ (0.02)	63.28^q^ (0.06)
52.5	700	55	9.67^k^ (0.01)	1.74^g^ (0.02)	4.99^j^ (0.01)	0.92^j^ (0.01)	27.47^k^ (0.06)	55.23^i^ (0.11)
52.5	800	45	7.15^c^ (0.02)	7.32^r^ (0.02)	8.98^p^ (0.03)	3.47^n^ (0.01)	31.26^o^ (0.06)	41.87^b^ (0.04)
47.5	700	45	9.14^i^ (0.04)	2.38^m^ (0.01)	4.62^h^ (0.01)	0.77^gh^ (0.01)	28.76^m^ (0.05)	54.34^g^ (0.03)
42.5	800	55	9.02^h^ (0.04)	2.52^n^ (0.01)	4.82^i^ (0.01)	0.80^hi^ (0.01)	26.07^hi^ (0.04)	56.79^k^ (0.11)
47.5	800	55	12.26^p^ (0.04)	0.47^a^ (0.00)	4.23^f^ (0.02)	0.18^a^ (0.04)	21.47^d^ (0.01)	61.41^o^ (0.01)
47.5	700	65	6.56^b^ (0.01)	10.26^t^ (0.02)	7.66^m^ (0.01)	5.51^p^ (0.01)	28.46^l^ (0.04)	41.57^a^ (0.06)
47.5	600	55	12.14^o^ (0.02)	0.52^b^ (0.01)	4.94^j^ (0.08)	0.24^b^ (0.02)	22.71^f^ (0.00)	59.46^n^ (0.07)
52.5	600	65	9.72^k^ (0.01)	1.61^f^ (0.01)	4.65^h^ (0.01)	0.82^i^ (0.01)	26.09^hi^ (0.01)	57.12^l^ (0.03)
42.5	800	65	9.83^l^ (0.03)	1.51^e^ (0.01)	3.04^b^ (0.01)	0.80^hi^ (0.01)	22.63^f^ (0.03)	62.20^p^ (0.06)
42.5	700	55	9.98^m^ (0.02)	1.41^d^ (0.02)	2.47^a^ (0.01)	0.63^d^ (0.02)	21.86^e^ (0.01)	63.67^r^ (0.03)

*X*_1_: feed moisture content; *X*_2_: screw speed rate; *X*_3_: barrel temperatures; MC: moisture content; CHO: carbohydrate. Standard deviations of triplicate measurement are represented in parentheses. Means with no common letters within a column significantly differ (*p *< 0.05).

There were also significance differences (*p* < 0.05) in the fat content of the cocoyam noodles with samples from FMC of 52.5%, SS of 800 rpm, and BT of 45°C having the highest fat content, while samples obtained from FMC of 42.5%, SS of 700 rpm, and BT of 55°C gave the lowest. This is quite important, as fats act as lubricants during extrusion cooking and reduces friction. Studies have also shown that extrusion cooking can cause structural and physicochemical changes, especially a redistribution of insoluble fiber to soluble ones (Castells et al., 2005; Gui et al., 2012). As such, SS and BT significantly (*p* < 0.05) affected the crude fiber of the noodles. The protein content of the extrudates was found to increase with increase in SS and/or BT. This could be attributed to degradation and denaturation of complex protein structures to smaller amino acids, with the changes more pronounced at higher SS (increased shear stress) and temperature conditions.

### 3.2 Thermo‐physical and extrudate properties

The thermo‐physical properties of the cocoyam noodles as influenced by the extrusion process variables are shown in Table 3. Bulk density gives an indication of the heaviness of the noodle samples and relative volume of packaging material required (Butt & Batool, 2010). Lower bulk densities are more preferred as this would translate to easier packaging and transportation (Adebiyi, Obadina, Mulaba‐Bafubiandi, Adebo, & Kayitesi, 2016; Agunbiade & Sanni, 2001). The obtained specific heat capacities of the cocoyam noodles were relatively high compared to the studies of Singh and Heldman (1993), Rapusas and Driscoll (1995) and Sobowale et al. (2014). Generally, the specific heat of a food may be influenced by the product properties (moisture content, temperature and pressure) and when high, the rate of energy conduction across/within the food sample is faster, and vice versa. However, in processing of pasta products, higher values of specific heat capacity usually lead to more energy transfer and improved heat transfer rate of the food sample (Baik & Mittal, 2003; Cengel, 1998).

**Table 3:** Thermo‐physical and extrudate properties of cocoyam noodles

Variables			*ρ*		*C*_p_		*K*_s_		*α*		RT		ER		MFR		CT	
*X*_1_ (%)	*X*_2_ (RPM)	*X*_3_ (°C)	Exp	Pred	Exp	Pred	Exp	Pred	Exp	Pred	Exp	Pred	Exp	Pred	Exp	Pred	Exp	Pred
52.5	800	65	1.13^d^ (0.01)	1.14	177.87^c^ (0.03)	178.00	23.99^b^ (0.07)	24.07	0.12^b^ (0.03)	0.12	52^a^ (0.03)	52.14	0.23^d^ (0.05)	0.23	2.35^a^ (0.07)	2.37	7.0^b^ (0.01)	7.79
42.5	600	55	1.25^e^ (0.03)	1.16	182.60^d^ (0.01)	184.69	24.13^d^ (0.02)	24.81	0.11^a^ (0.02)	0.12	55^b^ (0.01)	59.13	0.18^a^ (0.03)	0.20	2.42^c^ (0.04)	2.41	5.0^a^ (0.03)	4.92
52.5	600	45	0.96^b^ (0.06)	1.08	184.96^e^ (0.04)	182.86	24.45^d^ (0.01)	24.68	0.14^c^ (0.06)	0.13	60^d^ (0.07)	60.55	0.21^c^ (0.02)	0.19	2.38^a^ (0.01)	2.43	10.0^c^ (0.04)	10.47
42.5	800	45	1.25^e^ (0.02)	1.08	179.52^cd^ (0.02)	182.86	23.96^b^ (0.03)	24.68	0.11^a^ (0.04)	0.13	54^b^ (0.01)	60.55	0.17^a^ (0.01)	0.19	2.43^c^ (0.05)	2.43	8.0^b^ (0.02)	10.47
47.5	800	45	1.00^c^ (0.05)	1.07	187.09^f^ (0.01)	182.54	25.32^d^ (0.02)	24.24	0.14^c^ (0.07)	0.13	57^c^ (0.04)	58.64	0.22^b^ (0.01)	0.22	2.50^f^ (0.03)	2.46	14.0^e^ (0.01)	13.75
42.5	800	45	1.07^cd^ (0.03)	1.06	182.04^bc^ (0.08)	185.43	23.81^b^ (0.04)	24.73	0.12^b^ (0.03)	0.13	62^b^ (0.08)	62.54	0.20^b^ (0.08)	0.21	2.45^b^ (0.04)	2.46	10.0^c^ (0.02)	11.95
47.5	600	65	1.03^c^ (0.04)	1.07	185.07^e^ (0.02)	181.73	24.31^d^ (0.03)	24.03	0.13^b^ (0.05)	0.12	60^d^ (0.01)	59.31	0.22^c^ (0.02)	0.21	2.38^a^ (0.08)	2.41	7.0^b^ (0.04)	7.85
47.5	700	55	1.01^c^ (0.02)	0.98	160.78^a^ (0.02)	177.27	22.60^b^ (0.01)	24.50	0.14^c^ (0.06)	0.14	52^b^ (0.03)	58.19	0.21^c^ (0.07)	0.20	2.40^b^ (0.01)	2.42	7.0^b^ (0.01)	7.26
47.5	600	45	1.02^c^ (0.04)	1.10	181.36^d^ (0.03)	184.15	25.657^d^ (0.06)	25.31	0.14^c^ (0.02)	0.13	65^f^ (0.03)	62.21	0.22^b^ (0.02)	0.20	2.48^e^ (0.02)	2.46	12.0^d^ (0.02)	10.08
52.5	600	55	1.05^cd^ (0.03)	1.04	185.62^e^ (0.05)	187.57	26.00^e^ (0.04)	25.83	0.13^b^ (0.01)	0.13	64^b^ (0.02)	60.43	0.16^a^ (0.05)	0.19	2.47^d^ (0.05)	2.44	8.0^b^ (0.07)	8.29
52.5	700	55	1.00^c^ (0.06)	1.07	184.03^e^ (0.07)	175.65	24.70^b^ (0.02)	23.78	0.13^b^ (0.03)	0.12	58^c^ (0.01)	55.26	0.22^c^ (0.01)	0.20	2.42^c^ (0.01)	2.38	8.0^b^ (0.03)	6.64
52.5	800	45	1.23^e^ (0.02)	1.21	171.92^c^ (0.03)	177.39	21.86^a^ (0.03)	22.45	0.10^a^ (0.02)	0.10	54^b^ (0.07)	52.27	0.23^d^ (0.03)	0.23	2.37^a^ (0.04)	2.39	13.0^d^ (0.08)	12.53
47.5	700	45	1.15^bc^ (0.01)	1.07	182.54^d^ (0.02)	174.20	24.40^b^ (0.01)	23.36	0.12^b^ (0.04)	0.13	64^f^ (0.05)	59.24	0.20^b^ (0.02)	0.21	2.43^c^ (0.02)	2.42	12.0^d^ (0.01)	9.75
42.5	800	55	0.97^b^ (0.02)	0.92	181.72^d^ (0.04)	186.86	24.58^b^ (0.08)	25.84	0.14^c^ (0.06)	0.13	59^d^ (0.01)	60.57	0.21^c^ (0.07)	0.19	2.38^b^ (0.02)	2.46	10.0^c^ (0.05)	9.16
47.5	800	55	1.00^bc^ (0.01)	0.96	191.53^bc^ (0.02)	185.57	26.53^e^ (0.02)	25.85	0.14^c^ (0.09)	0.14	56^c^ (0.02)	57.75	0.18^a^ (0.09)	0.20	2.48^e^ (0.01)	2.46	10.0^c^ (0.01)	10.74
47.5	700	65	1.07^cd^ (0.01)	0.99	164.48^b^ (0.01)	171.71	21.21^a^ (0.04)	23.03	0.12^b^ (0.04)	0.14	53^a^ (0.08)	56.65	0.19^b^ (0.01)	0.22	2.33^b^ (0.08)	2.37	4.0^a^ (0.02)	6.48
47.5	600	55	1.01^c^(0.04)	1.04	191.57^bc^ (0.03)	187.26	26.28^e^ (0.05)	25.97	0.13^b^ (0.02)	0.13	66^g^ (0.01)	61.01	0.20^b^ (0.02)	0.19	2.45^d^ (0.06)	2.46	8.0^b^ (0.02)	8.11
52.5	600	65	1.12^d^ (0.06)	1.11	184.05^d^ (0.06)	183.64	24.92^d^ (0.07)	24.39	0.12^b^ (0.09)	0.12	59^d^ (0.02)	59.80	0.22^c^ (0.01)	0.21	2.43^c^ (0.01)	2.40	10.0^c^ (0.01)^c^	7.81
42.5	800	65	0.84^a^ (0.02)	0.88	183.26^d^ (0.03)	179.65	25.45^d^ (0.05)	24.36	0.16^e^ (0.01)	0.15	62^e^ (0.01)	58.10	0.21^c^ (0.05)	0.21	2.47^d^ (0.01)	2.40	10.0^c^ (0.08)	8.08
42.5	700	55	0.92^b^ (0.07)	1.03	183.63^d^ (0.04)	176.63	25.68^d^ (0.04)	23.91	0.15^d^ (0.05)	0.13	61^e^ (0.06)	58.66	0.21^c^ (0.04)	0.20	2.40^b^ (0.03)	2.39	4.0^a^ (0.01)	4.88

*X*_1_: feed moisture content; *X*_2_: screw speed rate; *X*_3_: barrel temperatures; *ρ*: density; *C*_p_: specific heat capacity; *K*_s_: thermal conductivity; *α*: thermal diffusivity; RT: residence time; ER: expansion ratio; MFR: mass flow rate; CT: cooking time; Exp: experimental value; Pred: Predicted value. Standard deviations of triplicate measurement are represented in parentheses. Means with no common letters within a column significantly differ (*p *˂ 0.05).

**Figure 1:**
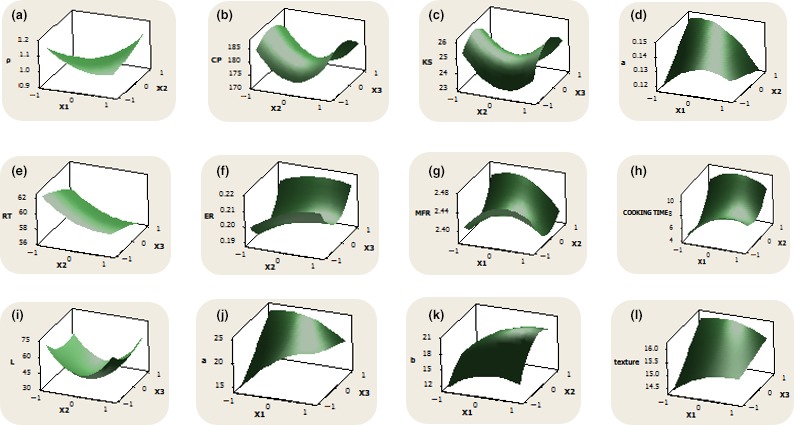
Surface plots of the responses evaluated (a) *ρ*—density, (b) *C*_P_—specific heat capacity, (c) *K*_s_—thermal conductivity, (d) *a*—thermal diffusivity, (e) RT—residence time, (f) ER—expansion ratio, (g) MFR—mass flow rate, (h) Cooking time, (i) *L*—lightness, (j) *a*—redness, (k) *b*—yellowness, (l) texture2017

The thermal diffusivity values obtained were ˂1, correlating well with other studies (Baik & Mittal, 2003; Nwanekezi & Ukagu, 1999; Sobowale et al., 2014). More so, the greater the density, the greater the contact between barrel surfaces, hence a corresponding higher thermal conductivity. With reference to the regression coefficient of the thermo‐physical properties (Table 4), only the interactive effect of feed moisture content and screw speed rate (*X*_1_ *X*_2_) had a significant effect (*p* < 0.05) on bulk density of the cocoyam noodles. Further representation of the model on the surface plot (Figure 1a) showed decrease in bulk density with increase in the FMC and SS rate. Other thermo‐physical parameters, specific heat capacity and thermal conductivity concurrently decreased with increase in SS rate and BT. On the other hand, increase in FMC and SS rate resulted in an increase in the thermal conductivity (Figure 1b–d).

**Table 4:** Coefficient of regression, *R* ^2^, AAD, *B*_f_, and *A*_f_ values for the mathematical models of the responses

Coeff	*ρ*	*C*_p_	*K*_s_	*α*	RT	ER	MFR	CT	L*	a*	b*	Texture
*α*_o_	0.98386	177.271	24.4986	0.138968	58.1939	0.198711	2.41916	7.2602	32.4399	22.6378	19.5520	15.6096
*α*_1_	0.02213	−0.491	−0.0646	−0.004423	−1.7039	0.001445	−0.00438	0.8775	−2.1080	1.4367	2.2846	0.1243
*α*_2_	−0.04035	−0.846	−0.0592	0.004473	−1.6331	0.005104	0.00296	1.3148*	0.8451	−0.1852	1.3429	−0.0403
*α*_3_	−0.03859	−1.249	−0.1639	0.003923	−1.2962	0.002479	−0.02116	−1.6349*	−6.5914	1.4596	1.6386	0.5319
*α*_11_	0.06661	−1.130	−0.6514	−0.010203	−1.2338	0.003091	−0.03285	−1.5027	2.3304	−2.0089	−1.8843	−0.2914
*α*_22_	0.01401	9.140	1.4133	−0.000151	1.1857	−0.004156	0.04138	2.1635	23.1889*	−6.3056	−3.3974	−1.5297
*α*_33_	0.04873	−4.316	−1.3000	−0.007217	−0.2508	0.015131	−0.02506	0.8520	8.6749	0.2743	−2.0007	0.0073
*α*_12_	0.08227*	−1.930	−0.5782	−0.010075	−2.3528	0.007816	−0.02262	−0.8058	1.5884	−0.1514	0.9601	−0.3925
*α*_13_	0.02427	1.596	0.4942	−0.002811	1.0756	0.002117	0.01009	−0.2162	9.4629	−3.4775	−6.6418*	−0.2976
*α*_23_	−0.02644	−0.041	0.4782	0.006507	0.1545	−0.003016	0.00308	−0.5200	2.2045	−1.7282	−2.1160	−0.3169
*R* ^2^	1.15	0.97	1.08	1.18	1.00	0.94	1.05	1.33	1.18	1.03	1.16	0.95
AAD	0.05	0.03	0.03	0.06	0.05	0.07	0.01	0.14	0.19	0.23	0.22	0.07
*B*_f_	1.00	1.00	1.00	1.00	1.00	1.00	1.00	1.02	1.02	1.04	1.08	1.00
*A*_f_	1.06	1.03	1.03	1.06	1.05	1.07	1.01	1.15	1.19	1.18	1.01	1.07

Coeff: coefficient; *ρ*: density; *C*_p_: specific heat capacity; *K*_s_: thermal conductivity; *α*: thermal diffusivity; RT: residence time; ER: expansion ratio; MFR: mass flow rate; CT: cooking time; L*: Lightness; a*: redness; b*: yellowness. *α*_0_, *α*_1_–*α*_3_, *α*_11_–*α*_33_, and *α*_12_–*α*_13_ are the equation regression coefficients for intercept, linear, quadratic, and interaction coefficient, respectively, AAD: average absolute deviation; *B*_f_: bias factor; *A*_f_: accuracy factor; *R* ^2^: coefficient of determination.

^*^Significant at *p < *0.05.

The extrudate properties of cocoyam noodles as influenced by extrusion process variables are presented in Table 3. The residence time (RT), expansion ratio (ER), mass flow rate (MFR), and cooking time (CT) of the cocoyam noodles ranged from 52.0 to 66.0 s, 0.16 to 0.23, 2.33 to 2.50 g/s, and 4 to 14 min, respectively. It has been reported that RT is a function of moisture content, feed rate, screw speed, barrel temperature, and screw geometry (Anuonye, Badifu, Inyang, & Akpapunam, 2007). Equally of significant importance is the specific mechanical energy which has been reported to influence product formation, geometry and expansion of the extrudate (Iwe et al., 2001). Results obtained from this study are in tandem with this assertion. The relationship between FMC, BT, and the RT was inversely proportional. This was expected because decreasing the moisture content of the feed under decreasing extruder BT would lead to slower dough melt and more plugging of extruder die, thus increasing the extrudate residency in the extruder barrel. This is equally reflected in the surface plot (Figure 1e), showing the impact of screw speed rate and barrel temperature on the residence time of the cocoyam noodles.

The ER of the extrudate sample increased with decrease in moisture content of the feeds. This is due to the fact that low moisture feeds exhibit more drag and therefore exert more pressure at the die, resulting into greater expansion at the exit of the die than for high moisture feeds (Arora, Zhao, & Camire, 1993; Bhattacharya, Kodiak, & Choudhury, 1994; Oluwole, 2008). Moisture is a major plasticizer in flours, which enables them to undergo glass transition during the extrusion process, facilitating matrix deformation and expansion. This study confirmed that SS generally has a positive effect (Figure 1f) on the expansion of the extrudates due to the increase in shear, subsequently leading to a decrease in melt viscosity induced by high SS (Ali, Hanna, & Chinnaswamy, 1996; Kokini, Chang, & Lai, 1992; Sobowale et al., 2016).

The extrudates samples with FMC of 47.5%, SS of 700 rpm and BT of 65°C had the lowest MFR, while the sample with FMC of 47.5%, SS of 800 rpm, and BT of 45°C had the highest MFR. Changes in the SS and BT did have significant effects on the MFR values, similar to the observation of other authors (Chevanan, Muthukumarappan, Rosentrater, & Julson, 2007; Choudhury & Gautam, 1999). Nonetheless, increases in the FMC and SS resulted in respective decrease and increase in MFR (Figure 1g). Higher SSs typically produce higher MFR due to the increased capability of the extruder screw to convey material through the extruder barrel. The relatively long CT observed in almost all the cocoyam noodle samples, thus suggests that the noodles during cooking will take up water more slowly. Accordingly, the regression coefficient of the model describing CT (Table 4) indicated that the positive and negative linear effects of SS and BT (*X*_2_ and *X*_3_ respectively) induced significant (*p* < 0.05) effects on the CT of the cocoyam noodles. The response surface plots (Figure 1h) equally revealed increases in CT as a result of increase in FMC, SS and decrease in BT of the cocoyam noodles.

### 3.3 Color and texture

The color [lightness (L*), redness (a*), and yellowness (b*)] attributes of the cocoyam noodles are shown in Table 5. The color attributes of the cocoyam noodles in terms of L*, a*, b* ranged from 29.38 to 77.18, 9.43 to 25.49, and 2.18 to 23.44, respectively. Acceptability of snack foods is mostly judged by color, with increase in FMC influencing increased lightness of the cocoyam noodles. This could also be related to the generally low values for bulk density and ER, resulting in a more porous and puffed noodles with better color. These parameters have earlier been observed to impart changes in the lightness of the extrudates (Chaiyakul, Jangchud, Jangchud, Wuttijumnong, & Winger, 2008; Joshi, Bera, & Panesar, 2014). Observations drawn from the coefficient of regression model (Table 4), indicated that the positive quadratic effect of SS ($X_{2}^{2}$ ) and negative quadratic interactive effects of FMC and BT (*X*_1_ *X*_3_) significantly (*p *< 0.05) influenced the L* and b* of the cocoyam noodles. A similar trend was also demonstrated on the surface plots (Figure 1I and 1K), wherein increases in the SS and FMC subsequently led to a decrease in the L* and increase in the b* of the noodles.

**Table 5:** Color and texture attributes of cocoyam noodles

Variables			L*		a*		b*		Texture	
*X*_1_ (%)	*X*_2_ (RPM)	*X*_3_ (°C)	Exp	Pred	Exp	Pred	Exp	Pred	Exp	Pred
52.5	800	65	74.88^p^ (0.01)	72.04	9.43^q^ (0.01)	11.95	11.66^n^ (0.01)	9.74	12.87^ab^ (0.15)	13.40
42.5	600	55	75.18^q^ (0.01)	60.81	9.44^q^ (0.01)	12.92	11.81^m^ (0.01)	11.60	12.20^a^ (1.74)	13.31
52.5	600	45	73.91^o^ (0.01)	61.43	10.86^n^ (0.01)	16.66	15.01^h^ (0.01)	15.14	12.77^ab^ (1.70)	13.80
42.5	800	45	73.66^n^ (0.01)	61.43	11.12^l^ (0.01)	16.66	14.81^i^ (0.01)	15.14	11.93^a^ (1.68)	13.80
47.5	800	45	77.18^t^ (0.01)	69.54	10.63^o^ (0.01)	16.69	12.13^l^ (0.01)	15.97	12.87^ab^ (0.15)	13.83
42.5	800	45	76.42^s^ (0.01)	81.85	11.01^m^ (0.01)	9.92	8.41^p^ (0.01)	4.20	13.07^ab^ (0.15)	13.51
47.5	600	65	65.99^l^ (0.01)	54.66	15.16^j^ (0.01)	19.98	18.45^f^ (0.01)	16.57	13.80^bc^ (0.30)	14.98
47.5	700	55	31.16^b^ (0.01)	32.44	19.46^g^ (0.01)	22.64	14.01^j^ (0.01)	19.55	15.07^cdef^ (0.12)	15.61
47.5	600	45	58.41^j^ (0.01)	72.25	19.86^f^ (0.01)	13.60	2.18^q^ (0.01)	9.06	15.07^cdef^ (0.23)	13.28
52.5	600	55	43.49^g^ (0.01)	53.42	21.58^e^ (0.01)	16.10	20.86^b^ (0.01)	14.25	14.80^cde^ (0.30)	14.35
52.5	700	55	32.86^d^ (0.01)	32.66	22.29^d^ (0.01)	22.07	18.49^e^ (0.01)	19.95	15.90^ef^ (0.20)	15.44
52.5	800	45	48.32^i^ (0.01)	61.88	25.49^a^ (0.01)	19.44	23.44^a^ (0.01)	23.98	15.00^cdef^ (0.10)	13.57
47.5	700	45	48.18^h^ (0.01)	47.71	25.46^b^ (0.01)	21.45	23.42^a^ (0.01)	15.91	16.17^f^ (0.21)	15.09
42.5	800	55	75.89^r^ (0.01)	59.32	9.58^p^ (0.01)	12.85	8.63^o^ (0.01)	12.37	14.00^bcd^ (0.10)	14.02
47.5	800	55	66.42^m^ (0.01)	56.47	14.89^k^ (0.01)	16.15	18.44^f^ (0.01)	17.50	12.97^ab^ (0.15)	14.04
47.5	700	65	42.63^f^ (0.01)	34.52	23.63^c^ (0.01)	24.37	20.41 (0.01)^c^	19.19	15.17^def^ (0.25)	16.15
47.5	600	55	32.41^c^ (0.01)	54.78	22.31^d^ (0.01)	16.52	19.52^d^ (0.01)	14.81	15.97^ef^ (0.21)	14.12
52.5	600	65	58.49^k^ (0.01)	62.76	18.19^i^ (0.01)	16.08	3.29^r^ (0.1)	9.36	16.00^ef^ (0.20)	14.90
42.5	800	65	36.14^e^ (0.01)	54.15	22.31^d^ (0.01)	16.34	17.58^g^ (0.01)	16.53	16.13^f^ (0.12)	14.54
42.5	700	55	29.38^a^ (0.16)	36.88	18.88^h^ (0.01)	19.19	13.66^k^ (0.01)	15.38	15.17^def^ (0.25)	15.19

*X*_1_: feed moisture content; *X*_2_: screw speed rate; *X*_3_: barrel temperatures; L*: Lightness; a*: redness; b*: yellowness; Exp: experimental value; Pred: Predicted value. Standard deviations of triplicate measurement are represented in parentheses. Means with no common letters within a column significantly differ (*p *˂ 0.05).

Texture is an important and desirable attribute of extruded products. The textural values of the cocoyam noodles ranged from 11.93 to 16.17 N. These values were observed to significantly (*p* ˂ 0.05) differ, as a function of FMC, SS and BT (Table 5). More significantly, higher temperature extrusion usually results into a product with more air cells, lighter (reduced wall thickness), and softer extrudates (Joshi et al., 2014). Coefficient of the regression models of the texture with respect to their linear, quadratic, or interaction effects did not show significant (*p* < 0.05) effect, but the influences of these process variables observed are shown on the surface plots (Figure 1l).

### 3.4 Physicochemical properties

The physicochemical properties of cocoyam noodles as influenced by FMC, BT, and SS are presented in Table 6. The WAC of the noodles sample varied between 0.87 and 1.84%, this trend in values could be attributed to the different extruding conditions. According to Niba, Bokanga, Jackson, Schlimme, and Li (2001), WAC depends on the availability of hydrophilic groups that bind water molecules and has been used to estimate the suitability, bulkiness, and consistency of extrudates (Oluwole, 2008). SC of the sample generally indicates the level of starch content and the extent of gelatinization of inherent starch. Insufficient water uptake which is directly related to swelling usually results in noodles with hard and coarse texture, but excess water uptake has been linked to noodles that are too soft (Petitot, Boyer, Minier, & Micard, 2010). Water solubility equally gives information about degradation of starch granules while water absorption is more related to the swelling capability of the sample. Samples obtained from feed with FMC of 42.5%, SS of 800 rpm and BT of 45°C had the lowest solubility index, while sample from FMC of 52.5%, SS of 600 rpm, and BT of 55°C gave the highest.

**Table 6:** Physiochemical properties of cocoyam noodles

Variables			WAC		pH		SC		SI		AC		APC	
*X*_1_ (%)	*X*_2_ (RPM)	*X*_3_ (°C)	Exp	Pred	Exp	Pred	Exp	Pred	Exp	Pred	Exp	Pred	Exp	Pred
52.5	800	65	0.90^bc^ (0.01)	1.07	6.45^gh^ (0.01)	6.44	2.62^d^ (0.01)	3.32	2.35^e^ (0.04)	2.85	26.18^f^ (0.01)	30.30	73.82^k^ (0.01)	69.70
42.5	600	55	0.98^e^ (0.01)	1.09	6.39^b^ (0.01)	6.43	3.3^i^ (0.01)	3.45	2.99^i^ (0.01)	3.28	25.52^e^ (0.01)	29.32	74.48^s^ (0.01)	70.68
52.5	600	45	0.96^d^ (0.01)	1.41	6.50^k^ (0.00)	6.48	2.81^e^ (0.01)	4.07	2.48^f^ (0.02)	3.45	44.06^o^ (0.01)	38.45	55.94^a^ (0.01)	61.55
42.5	800	45	1.13^g^ (0.01)	1.41	6.44^fgh^ (0.01)	6.48	3.24^g^ (0.03)	4.07	2.87^g^ (0.02)	3.45	23.19^c^ (0.01)	38.45	76.81^i^ (0.01)	61.55
47.5	800	45	1.09^f^ (0.01)	1.18	6.43^f^ (0.01)	6.40	3.19^f^ (0.01)	3.22	2.86^g^ (0.04)	2.62	50.05^q^ (0.01)	48.15	49.95^n^ (0.01)	51.85
42.5	800	45	0.87^a^ (0.01)	0.87	6.37^a^ (0.01)	6.40	2.12^a^ (0.03)	2.02	1.11^a^ (0.01)	1.04	48.28^p^ (0.01)	39.34	51.72^j^ (0.01)	60.66
47.5	600	65	0.89^ab^ (0.01)	1.36	6.39^d^ (0.00)	6.45	2.48^c^ (0.01)	3.93	2.25^d^ (0.04)	3.93	28.58^g^ (0.01)	33.14	71.42^b^ (0.01)	66.86
47.5	700	55	1.13^g^ (0.01)	1.40	6.43^fg^ (0.00)	6.44	3.29^h^ (0.01)	4.22	2.93^ghi^ (0.04)	3.17	40.32^m^ (0.01)	34.71	59.68^h^ (0.01)	65.29
47.5	600	45	1.53^j^ (0.01)	1.15	6.44^fgh^ (0.00)	6.44	4.45^m^ (0.01)	3.14	3.90^kl^ (0.00)	2.84	50.29^r^ (0.01)	37.72	49.71^t^ (0.01)	62.28
52.5	600	55	1.84^m^ (0.01)	1.42	6.41^e^ (0.01)	6.47	5.45^r^ (0.0.2)	4.43	4.83^o^ (0.04)	3.70	25.21^d^ (0.01)	20.50	74.79^i^ (0.01)	79.50
52.5	700	55	1.56^k^ (0.01)	1.49	6.44^c^ (0.01)	6.45	5.39^q^ (0.01)	4.69	2.97^hi^ (0.03)	3.32	38.43^l^ (0.01)	30.30	61.57^d^ (0.01)	69.70
52.5	800	45	1.69^l^ (0.01)	1.39	6.44^fgh^ (0.00)	6.41	4.95^o^ (0.02)	4.26	4.42^m^ (0.01)	3.67	56.42^t^ (0.01)	52.93	43.58^t^ (0.01)	47.07
47.5	700	45	1.43^h^ (0.01)	1.29	6.41^e^ (0.01)	6.42	3.55^j^ (0.01)	3.52	1.81^c^ (0.01)	2.38	29.19^h^ (0.01)	46.44	70.81^h^ (0.01)	53.56
42.5	800	55	0.92^c^ (0.01)	1.06	6.52^l^ (0.01)	6.45	2.29^b^ (0.02)	3.07	1.23^b^ (0.01)	2.36	19.52^b^ (0.01)	33.83	80.48^b^ (0.01)	66.17
47.5	800	55	0.97^de^ (0.01)	1.23	6.39^de^ (0.01)	6.44	3.29^h^ (0.01)	3.76	2.92^ghi^ (0.02)	3.27	31.14^j^ (0.01)	35.49	68.86^j^ (0.01)	64.51
47.5	700	65	1.55^k^ (0.00)	1.38	6.47^i^ (0.01)	6.46	4.53^n^ (0.01)	3.96	3.92^l^ (0.09)	3.19	42.77^n^ (0.01)	39.99	57.23^n^ (0.01)	60.01
47.5	600	55	1.71^l^ (0.02)	1.31	6.53^l^ (0.01)	6.45	4.99^p^ (0.01)	4.02	4.56^n^ (0.03)	3.76	30.22^i^ (0.01)	26.93	69.78^i^ (0.01)	73.07
52.5	600	65	1.43^h^ (0.01)	1.32	6.49^j^ (0.01)	6.45	4.20^k^ (0.03)	3.81	3.73^j^ (0.06)	3.20	17.01^a^ (0.01)	19.56	82.99^a^ (0.01)	80.44
42.5	800	65	1.50^i^ (0.01)	1.14	6.50^k^ (0.01)	6.50	4.35^l^ (0.01)	3.16	3.83^k^ (0.02)	2.92	53.78^s^ (0.01)	45.34	46.22^s^ (0.01)	54.66
42.5	700	55	1.10^f^ (0.00)	1.21	6.45^h^ (0.00)	6.44	3.23^fg^ (0.03)	3.60	2.90^gh^ (0.09)	2.47	35.80^k^ (0.01)	35.08	64.20^k^ (0.01)	64.93

*X*_1_: feed moisture content; *X*_2_: screw speed rate; *X*_3_: barrel temperatures; WAC: water absorption capacity; SC: swelling capacity; SI: solubility index; AC: amylose content; APC: amylopectin content; Exp: experimental value; Pred: Predicted value. Standard deviations of triplicate measurement are represented in parentheses. Means with no common letters within a column significantly differ (*p *˂ 0.05).

**Figure 2:**
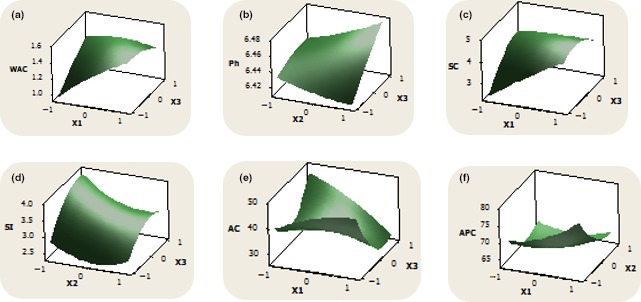
Surface plots of the responses evaluated (a) WAC—water absorption capacity, (b) pH, (c) SC—swelling capacity, (d) SI—solubility index, (e) AC—amylose content, (f) APC—amylopectin content

The amylose and amylopectin content of noodles sample ranged between 17.01%–56.42% and 43.58%–82.99%, respectively. Samples from FMC of 52.5%, SS of 600 rpm, and BT of 65°C had the lowest amylose content, while the sample from FMC of 52.5%, SS of 800 rpm, and BT of 45°C gave the highest and vice versa in the case of amylopectin content. High BT during extrusion cooking promotes starch gelatinization and reduces amylose leaching in the cooking water (Adedotun, Adebowale, Olayiwola, Shittu, & Sanni, 2015). This study showed that there were significantly differences (*p *˂ 0.05) in the all the physicochemical properties of the cocoyam noodles. Nonetheless, the linear, quadratic, and interaction effects of the regression model representing these physiochemical properties were not significant (*p* < 0.05), although the surface plots (Figure 2a–f) showed some trend.

### 3.5 Validation of statistical models

The effects of three independent extrusion variables feed moisture content (*X*_1_), screw speed rate (*X*_2_), barrel temperature (*X*_3_) on responses [Density (*ρ*), specific heat capacity (*C*_p_), thermal conductivity (*K*_s)_, thermal diffusivity (*α*), lightness (L*), redness (a*), yellowness (b*), texture, residence time (RT), expansion ratio (ER), mass flow rate (MFR), cooking time (CT), pH, water absorption capacity (WAC), swelling capacity (SC), solubility index (SI), amylose content (AC), and amylopectin content (APC)] which were investigated in this study, and the different models representing each are provided in Equations (10), (11), (12), (13), (14), (15), (16), (17), (18), (19), (20), (21), (22), (23), (24), (25), (26), (27).

(10) $$\rho \begin{array}{cc} = & 0.98386+0.02213X_{1}-0.04035X_{2}-0.03895X_{3}\\ & +0.06661X_{2}^{1}+0.01401X_{2}^{2}+0.04873X_{2}^{3}+0.08227X_{1}X_{2}\\ & +0.02427X_{1}X_{3}-0.02644X_{2}X_{3} \end{array}$$

(11) $$C_{p}\begin{array}{cc} = & 177.271-0.491X_{1}-0.846X_{2}-1.249X_{3}-1.130X_{2}^{1}\\ & +9.140X_{2}^{2}-4.316X_{2}^{3}-1.930X_{1}X_{2}+1.596X_{1}X_{3}-0.041X_{2}X_{3} \end{array}$$

(12) $$K_{s}\begin{array}{cc} = & 24.4986-0.0646X_{1}-0.0592X_{2}-0.1639X_{3}\\ & -0.6514X_{2}^{1}+1.4133X_{2}^{2}-1.3000X_{2}^{3}\\ & -0.5782X_{1}X_{2}+0.4942X_{1}X_{3}+0.4782X_{2}X_{3} \end{array}$$

(13) $$\alpha \begin{array}{cc} = & 0.138968-0.004423X_{1}+0.004473X_{2}\\ & +0.003923X_{3}-0.010203X_{2}^{1}-0.000151X_{2}^{2}\\ & -0.007217X_{2}^{3}-0.010075X_{1}X_{2}-0.002811X_{1}X_{3}+0.006507X_{2}X_{3} \end{array}$$

(14) $$L^{*}\begin{array}{cc} = & 32.4399-2.1080X_{1}+0.8451X_{2}-6.5914X_{3}\\ & +2.3304X_{2}^{1}+23.1889X_{2}^{2}+8.6749X_{2}^{3}+1.5884X_{1}X_{2}\\ & +9.4629X_{1}X_{3}+2.2045X_{2}X_{3} \end{array}$$

(15) $$a^{*}\begin{array}{cc} = & 22.6378+1.4367X_{1}-0.1852X_{2}+1.4596X_{3}\\ & -2.0089X_{2}^{1}-6.3056X_{2}^{2}+0.2743X_{2}^{3}\\ & -0.1514X_{1}X_{2}-3.4775X_{1}X_{3}-1.7282X_{2}X_{3} \end{array}$$

(16) $$b^{*}\begin{array}{cc} = & 19.5520+2.2846X_{1}+1.3429X_{2}+1.6386X_{3}\\ & -1.8843X_{2}^{1}-3.3974X_{2}^{2}-2.0007X_{2}^{3}\\ & +0.9601X_{1}X_{2}-6.6418X_{1}X_{3}-2.1160X_{2}X_{3} \end{array}$$

(17) $$\text{Texture}\begin{array}{cc} = & 15.6096+0.1243X_{1}-0.0403X_{2}+0.5319X_{3}\\ & -0.2914X_{2}^{1}-1.5297X_{2}^{2}+0.0073X_{2}^{3}-0.3925X_{1}X_{2}\\ & -0.2976X_{1}X_{3}-0.3169X_{2}X_{3} \end{array}$$

(18) $$\text{RT}\begin{array}{cc} = & 58.1939-1.7039X_{1}-1.6331X_{2}-1.2962X_{3}\\ & -1.2338X_{2}^{1}+1.1857X_{2}^{2}-0.2508X_{2}^{3}-2.3528X_{1}X_{2}\\ & +1.0756X_{1}X_{3}+0.1545X_{2}X_{3} \end{array}$$

(19) $$\text{ER}\begin{array}{cc} = & 0.198711+0.001445X_{1}+0.005104X_{2}+0.002479X_{3}\\ & +0.003091X_{2}^{1}-0.004156X_{2}^{2}+0.015131X_{2}^{3}\\ & +0.007816X_{1}X_{2}+0.002117X_{1}X_{3}-0.003016X_{2}X_{3} \end{array}$$

(20) $$\text{MFR}\begin{array}{cc} = & 2.41916-0.00438X_{1}+0.00296X_{2}0.02116X_{3}\\ & -0.03285X_{2}^{1}+0.04138X_{2}^{2}-0.02506X_{2}^{3}\\ & -0.02262X_{1}X_{2}+0.01009X_{1}X_{3}+0.00308X_{2}X_{3} \end{array}$$

(21) $$\text{CT}\begin{array}{cc} = & 7.2602+0.8775X_{1}+1.3148X_{2}-1.6349X_{3}\\ & -1.5027X_{2}^{1}+2.1635X_{2}^{2}+0.8520X_{2}^{3}\\ & -0.8058X_{1}X_{2}-0.2162X_{1}X_{3}-0.5200X_{2}X_{3} \end{array}$$

(22) $$\text{WAC}\begin{array}{cc} = & 1.39833+0.13952X_{1}-0.04140X_{2}+0.04534X_{3}\\ & -0.05175X_{2}^{1}-0.12920X_{2}^{2}-0.05908X_{2}^{3}\\ & -0.02623X_{1}X_{2}-0.14666X_{1}X_{3}-0.05868X_{2}X_{3} \end{array}$$

(23) $$\text{pH}\begin{array}{cc} = & 6.44169+0.00548X_{1}+0.00389X_{2}+0.01898X_{3}\\ & +0.00036X_{2}^{1}+0.00327X_{2}^{2}-0.00458X_{2}^{3}-0.01773X_{1}X_{2}\\ & -0.01781X_{1}X_{3}+0.01408X_{2}X_{3} \end{array}$$

(24) $$\text{SC}\begin{array}{cc} = & 4.22406+0.54617X_{1}-0.13155X_{2}\\ & +0.22029X_{3}-0.08124X_{2}^{1}-0.33684X_{2}^{2}-0.48391X_{2}^{3}\\ & +0.05748X_{1}X_{2}-0.51979X_{1}X_{3}-0.16999X_{2}X_{3} \end{array}$$

(25) $$\text{SI}\begin{array}{cc} = & 3.1659+0.4270X_{1}-0.2468X_{2}+0.4055X_{3}\\ & -0.2693X_{2}^{1}+0.3488X_{2}^{2}-0.3804X_{2}^{3}\\ & +0.2143X_{1}X_{2}-0.6746X_{1}X_{3}-0.1401X_{2}X_{3} \end{array}$$

(26) $$\text{AC}\begin{array}{cc} = & 34.7077-2.3850X_{1}+4.2803X_{2}-3.2242X_{3}\\ & -2.0177X_{2}^{1}-3.4990X_{2}^{2}+8.5033X_{2}^{3}\\ & +2.0232X_{1}X_{2}-7.1570X_{1}X_{3}-0.9324X_{2}X_{3} \end{array}$$

(27) $$\text{APC}\begin{array}{cc} = & 65.2923+2.3850X_{1}-4.2803X_{2}+3.2242X_{3}\\ & +2.0177X_{2}^{1}+3.4990X_{2}^{2}-8.5033X_{2}^{3}\\ & -2.0232X_{1}X_{2}+7.1570X_{1}X_{3}+0.9324X_{2}X_{3} \end{array}$$

The computed coefficients of determination (*R* ^2^) were greater than 0.85, implying a better consonance between the experimental and predicted values (Tables 4 and 7). Previous studies have affirmed that good fit of empirical model and experimental data is depicted by *R* ^2^ > 0.80 (Adebo et al., 2018; Odunmbaku et al., 2018; Sobowale, Ayodeji, & Adebiyi, 2017). Apart from high *R* ^2^ values which indicated validity of the model, other measures including bias factor (*B*_f_) and accuracy factor (*A*_f_) judged by nearness to unity (1) and not excluding average absolute deviation (AAD) (values close to zero), all gave acceptable results between the estimated (predicted) and actual data (experimental) (Adebo et al., 2018; Sobowale, Adebiyi, & Adebo, 2017). The ranges of these values obtained in this study further indicate the adequacy of the models for describing the investigated parameters.

**Table 7:** Coefficient of regression, *R* ^2^, AAD, *B*_f_, and *A*_f_ values for the mathematical models of the responses

Coefficient	WAC	Ph	SC	SI	AC	APC
*α*_o_	1.39833	6.44169	4.22406	3.1659	34.7077	65.2923
*α*_1_	0.13952	0.00548	0.54617	0.4270	−2.3850	2.3850
*α*_2_	−0.04140	0.00389	−0.13155	−0.2468	4.2803	−4.2803
*α*_3_	0.04534	0.01898	0.22029	0.4055	−3.2242	3.2242
*α*_11_	−0.05175	0.00036	−0.08124	−0.2693	−2.0177	2.0177
*α*_22_	−0.12920	0.00327	−0.33684	0.3488	−3.4990	3.4990
*α*_33_	−0.05908	−0.00458	−0.48391	−0.3804	8.5033	−8.5033
*α*_12_	−0.02623	−0.01773	0.05748	0.2143	2.0232	−2.0232
*α*_13_	−0.14666	−0.01781	−0.51979	−0.6746	−7.1570	7.1570
*α*_23_	−0.05868	0.01408	−0.16999	−0.1401	−0.9324	0.9324
*R* ^2^	0.85	0.89	0.94	1.01	1.12	1.12
AAD	0.19	0.00	0.20	0.21	0.21	0.11
*B*_f_	1.02	1.00	1.02	1.03	1.03	1.01
*A*_f_	1.20	1.00	1.21	1.23	1.22	1.11

WAC: water absorption capacity; SC: swelling capacity; SI: solubility index; AC: amylose content; APC: amylopectin content. *α*_0_, *α*_1_–*α*_3_, *α*_11_–*α*_33_, and *α*_12_–*α*_13_ are the equation regression coefficients for intercept, linear, quadratic, and interaction coefficient, respectively, AAD: average absolute deviation; *B*_f_: bias factor; *A*_f_: accuracy factor; *R* ^2^: coefficient of determination.

### 3.6 Sensory properties

Table 8 shows the sensory scores of the cocoyam noodles as affected by extrusion process variables. These were appearance, taste, chewiness, glossiness, firmness, and overall acceptability. The appearance of cocoyam noodles ranged between the mean scores of 3.35 and 7.75, the taste ranged between 2.15 and 7.95, mean score of chewiness ranged between 1.65 and 8.10, glossiness ranged between 2.20 and 8.40, firmness ranged between 2.55 and 7.65, while the overall acceptability of the sample ranged between 4.30 and 8.50. With respect to overall acceptability, the extrudates (cocoyam noodles) produced from FMC of 47.5%, SS of 700 rpm, and BT of 55°C gave the highest mean score (8.50) and were most preferred, while sample obtained from FMC of 52.5%, SS of 800 rpm, and BT of 45°C had the lowest score (4.25) and were least preferred.

**Table 8:** Sensory properties of cocoyam noodles

Variables			APP	Taste	Chewiness	Glossiness	Firmness	Overall acceptability
*X*_1_ (%)	*X*_2_ (RPM)	*X*_3_ (°C)						
52.5	800	65	5.85^def^	5.20^e^	5.90^efg^	5.05^f^	4.30^b^	4.55^a^
42.5	600	55	5.80^def^	5.50^ef^	6.20^g^	5.75^gh^	6.80^g^	7.00^de^
52.5	600	45	3.60^a^	3.55^b^	3.60^c^	2.20^a^	2.55^a^	4.30^a^
42.5	800	45	5.15^c^	5.20^e^	5.90e^fg^	4.40^e^	5.75^de^	5.80^bc^
47.5	800	45	6.25^ef^	6.10^fg^	5.25^e^	6.00^ghi^	6.30^efg^	6.40^cd^
42.5	800	45	5.70^de^	6.20^gh^	5.70^efg^	2.80^b^	4.20^b^	4.90^a^
47.5	600	65	6.25^ef^	6.10^fg^	5.25^e^	6.00^ghi^	6.30^efg^	6.40^cd^
47.5	700	55	7.75^g^	7.95^i^	8.10^i^	8.40^k^	7.65^h^	8.50^f^
47.5	600	45	5.85^def^	5.20^e^	5.90^efg^	5.05^f^	4.30^b^	4.55^a^
52.5	600	55	3.35_a_	4.90_de_	2.50_b_	3.45_cd_	2.10_a_	4.30_a_
52.5	700	55	5.40^cd^	4.00^bc^	6.05^fg^	5.65^fg^	5.25^cd^	5.65^b^
52.5	800	45	4.25^b^	2.15^a^	1.65^a^	3.05^bc^	5.15^c^	4.25^a^
47.5	700	45	6.00^ef^	6.10^fg^	6.30^g^	5.70^gh^	6.90^g^	6.35^cd^
42.5	800	55	5.80^de^	6.70^gh^	6.00^fg^	7.05^j^	6.90^g^	7.00^de^
47.5	800	55	6.30^f^	6.25^gh^	6.95^h^	6.45^i^	6.75^fg^	7.35^e^
47.5	700	65	6.25^ef^	6.10^fg^	5.25^e^	6.00^ghi^	6.30^efg^	6.40^cd^
47.5	600	55	5.75^def^	6.80^h^	5.45^ef^	5.75^gh^	6.15^ef^	7.00^de^
52.5	600	65	5.00^c^	4.35^cd^	4.40^d^	3.85^de^	5.25^cd^	6.25^bc^
42.5	800	65	6.20^ef^	6.65^gh^	6.10^fg^	6.35^hi^	6.35^efg^	6.35^cd^
42.5	700	55	6.25^ef^	6.15^g^	5.30^e^	6.00^ghi^	6.30^efg^	6.45^cd^

*X*_1_: feed moisture content; *X*_2_: screw speed rate; *X*_3_: barrel temperatures; APP: appearance. Standard deviations of triplicate measurement are represented in parentheses. Means with no common letters within a column significantly differ (*p *< 0.05).

Raw food materials undergo physical and chemical modifications such as gelatinization, breakdown of starch, denaturation of proteins, and interactions between them resulting from high temperatures and pressures with combined of shearing effect during extrusion. These changes affected the sensory properties such as appearance, taste, chewiness, glossiness, firmness and overall acceptability of the extruded products. These were reflected in the significant differences (*p *˂ 0.05) in all the sensory score obtained for the cocoyam noodles. Accordingly, these sensory properties are important for extruded food products being developed as new entrants into the market.

## 4 CONCLUSION

The study generally revealed that varying the feed moisture content, barrel temperature, and screw speed significantly (*p *< 0.05) affected the quality (proximate, thermo‐physical, physicochemical, color, and textural properties), extrudate properties and sensory characteristics of cocoyam noodles. The combined effect of feed moisture content at 47.5%, screw speed of 700 rpm and barrel temperature of 55°C gave the optimal extrusion process conditions for the production of quality cocoyam noodles. Not only was the extrudate at this condition the most preferred by the sensory panelists, it equally had desirable values of the investigated parameters. These variables are important considerations for commercial and mass production of healthy and nutritious instant extruded cocoyam noodles and thus positions extrusion as a viable alternative for transforming cocoyam into a value‐added product.

## CONFLICT OF INTEREST

The authors declare that we do not have any conflict of interest.

## ETHICAL REVIEW

This study was approved by the Polytechnic ethical committee.

## INFORMED CONSENT

Written informed consent was obtained all study participants.

## References

[fsn3786-bib-0001] Adebiyi, J. A. , Obadina, A. O. , Mulaba‐Bafubiandi, A. F. , Adebo, O. A. , & Kayitesi, E. (2016). Effect of fermentation and malting on the microstructure and selected physicochemical properties of pearl millet (*Pennisetum glaucum*) flour and biscuit. Journal of Cereal Science, 70, 132–139. 10.1016/j.jcs.2016.05.026

[fsn3786-bib-0002] Adebo, O. A. , Njobeh, P. B. , Mulaba‐Bafubiandi, A. F. , Adebiyi, J. A. , Desobgo, S. C. Z. , & Kayitesi, E. (2018). Optimization of fermentation conditions for ting production using response surface methodology. Journal of Food Processing and Preservation, 42, e13381 10.1111/jfpp.13381

[fsn3786-bib-0003] Adedotun, H. , Adebowale, A. A. , Olayiwola, I. O. , Shittu, T. A. , & Sanni, L. O. (2015). Production and quality evaluation of noodles from sweet potato starch. Journal of Culinary Science and Technology, 13, 79–93. 10.1080/15428052.2014.952479

[fsn3786-bib-0004] Agunbiade, S. O. , & Sanni, M. O. (2001). The effect of ambient storage of cassava tuber on starch quality In Root Crop. The small processor and development of local food industries for market economy. Proceeding on the eight triennial symposium of the international society for tropical root crops, Africa branch (ISTRC –AB). L 12‐16 NOV. 2001. IITA, Ibadan, Nigeria,189–194.

[fsn3786-bib-0005] Ali, Y. , Hanna, M. A. , & Chinnaswamy, R. (1996). Expansion characteristics of extruded corn grits. Lebensmittel‐Wissenschaft and Technologie, 29, 702–707. 10.1006/fstl.1996.0109

[fsn3786-bib-0006] Anuonye, J. C. , Badifu, G. I. O. , Inyang, C. U. , & Akpapunam, M. A. (2007). Effect of extrusion process variables on the amylase and pasting characteristics of acha/soybean extrudates using response surface analysis. American Journal of Food Technology, 2, 354–365.

[fsn3786-bib-0008] AOAC (2006). Official methods of analysis. Arlington, VA: Association Official Analytical Chemists.

[fsn3786-bib-0009] Arora, A. , Zhao, J. , & Camire, M. E. (1993). Extruded potato peels functional properties affected by extrusion conditions. Journal of Food Science, 58, 335–337. 10.1111/j.1365-2621.1993.tb04269.x

[fsn3786-bib-0010] Baik, O. D. , & Mittal, G. S. (2003). Kinetics of *tofu* colour change during deep‐fat frying. Lebensmittel‐Wissenschaft and Technologie, 36, 43–48. 10.1016/S0023-6438(02)00175-5

[fsn3786-bib-0011] Bhattacharya, S. , Kodiak, A. K. , & Choudhury, G. S. (1994). Twin‐screw extrusion of rice flour: Effect of extruder length‐to‐diameter ratio and barrel temperature on extrusion parameters and product characteristics. Journal of Food Processing and Preservation, 18, 389–406. 10.1111/j.1745-4549.1994.tb00261.x

[fsn3786-bib-0012] Bown, D. (2000). Aroids plants of the Arum family (2nd ed., p. 392). Portland, OR: Timber Press.

[fsn3786-bib-0013] Butt, M. S. , & Batool, R. (2010). Nutritional and functional properties of some promising legumes protein isolates. Pakistan Journal of Nutrition, 9, 373–379.

[fsn3786-bib-0014] Castells, M. , Marin, S. , Sanchis, V. , & Ramos, A. J. (2005). Fate of mycotoxins in cereals during extrusion cooking: A review. Food Additives and Contamination, 22, 150–157. 10.1080/02652030500037969 15824005

[fsn3786-bib-0015] Cengel, Y. (1998). Heat transfer—A practical approach. New York, NY: Mc Graw Hill.

[fsn3786-bib-0016] Chaiyakul, S. , Jangchud, K. , Jangchud, A. , Wuttijumnong, P. , & Winger, R. (2008). Effect of protein content and extrusion process on sensory and physical properties of extruded high‐protein, glutinous rice‐based snack. Kasetsart Journal of Natural Science, 7, 145–150.

[fsn3786-bib-0017] Changi, Y. K. , Martinez‐Bustos, F. , & Larai, H. (1998). Effect of some extrusion variables on rheological properties and physicochemical changes of cornmeal extruded by twin screw extruder. Brazilian Journal of Chemical Engineering, 15, 796–801.

[fsn3786-bib-0018] Chevanan, N. , Muthukumarappan, K. , Rosentrater, K. A. , & Julson, J. L. (2007). Effect of die dimensions on extrusion processing parameters and properties of DDGS. Cereal Chemistry, 84, 389–398. 10.1094/CCHEM-84-4-0389

[fsn3786-bib-0019] Choudhury, G. S. , & Gautam, A. (1999). Screw configuration effects on macroscopic characteristics of extrudates produced by twin‐screw extrusion of rice flour. Journal of Food Science, 64, 479–487. 10.1111/j.1365-2621.1999.tb15067.x

[fsn3786-bib-0020] Da Silva, P. , & Moreira, R. (2008). Vacuum frying of high quality fruit and vegetable based snacks. Lebensmittel‐Wissenschaft and Technologie‐Food Science and Technology, 41, 1758–1767. 10.1016/j.lwt.2008.01.016

[fsn3786-bib-0021] Emmanuel‐Ikpemel, C. A. , Eneji, C. A. , & Essiet, U. (2007). Storage stability and sensory evaluation of taro chips fried in palm oil, palm olein oil, groundnut oil, soybean oil and their blends. Pakistan Journal of Nutrition, 6, 570–575.

[fsn3786-bib-0022] FAOSTAT (Food and Agriculture Organization of the United Nations Statistics) (2016). Production data for crops. Retrieved from http://www.fao.org/faostat/en/#data/QC (Accessed 20/12/2016).

[fsn3786-bib-0023] Filli, K. B. , Jideani, A. I. O. , & Jideani, V. A. (2014). Extrusion bolsters food security in Africa. Food Technology, 68, 45–55.

[fsn3786-bib-0024] Gui, Y. , Gil, S. K. , & Ryu, G. H. (2012). Effects of extrusion conditions on the physicochemical properties of extruded red ginseng. Preventive Nutrition and Food Science, 17, 203–209. 10.3746/pnf.2012.17.3.203 24471085 PMC3866741

[fsn3786-bib-0025] Hussain, A. M. , & Rahman, M. S. (1999). Thermal conductivity prediction of fruit and vegetables using neural networks. International Journal of Food Properties, 2, 51–60.

[fsn3786-bib-0026] Iwe, M. O. , Vanzuilichem, D. J. , & Ngoddy, P. O. (2001). Extrusion cooking of blends of soy flour and sweet potato flour on specific mechanical energy (SME), extrudate temperature and torque. Journal of Food Processing and Preservation, 4, 251–266. 10.1111/j.1745-4549.2001.tb00459.x

[fsn3786-bib-0027] Joshi, S. M. R. , Bera, M. B. , & Panesar, P. S. (2014). Extrusion cooking of maize/spirulina mixture: Factors affecting expanded product characteristics and sensory quality. Journal of Food Processing and Preservation, 38, 655–664. 10.1111/jfpp.12015

[fsn3786-bib-0028] Kaushal, P. , Kumar, V. , & Sharma, H. K. (2012). Comparative study of physicochemical, functional, antinutritional and pasting properties of taro (*Colocasia esculenta*), rice (*Oryza sativa*) flour, pigeonpea (*Cajanus cajan*) flour and their blends. Lebensmittel‐Wissenschaft and Technologie, 48, 59–68. 10.1016/j.lwt.2012.02.028

[fsn3786-bib-0029] Kokini, J. L. , Chang, C. N. , & Lai, L. S. (1992). The role of rheological properties on extrudate expansion In Kokini J. L., Ho C.‐T., & Karwe M. V. (Eds.), Food extrusion science and technology (pp. 631–653). New York, NY: Marcel Dekker Incorporation.

[fsn3786-bib-0030] Korkerd, S. , Wanlapa, S. , Puttanlek, C. , Uttapap, D. , & Rungsardthong, V. (2016). Expansion and functional properties of extruded snacks enriched with nutrition sources from food processing by‐products. Journal of Food Science and Technology, 53, 561–570. 10.1007/s13197-015-2039-1 26787975 PMC4711464

[fsn3786-bib-0031] Kure, O. A. , Bahago, E. J. , & Daneil, E. A. (1998). Studies on the proximate composition and effect of particles size on acceptability of biscuit produced from blends of soyabean and plantain flours. Namoda Tech‐Scope Journal, 3, 17–21.

[fsn3786-bib-0032] Niba, L. L. , Bokanga, M. , Jackson, F. I. , Schlimme, D. S. , & Li, B. W. (2001). Physico‐chemical properties and starch granular characteristics of flour from various *Manihot esculenta* (cassava). Genotypes Journal, 67, 1701.

[fsn3786-bib-0033] Nwanekezi, E. C. , & Ukagu, J. C. (1999). Determination of engineering properties of some Nigerian fruits and vegetables. Nigerian Food Journal, 17, 55–59.

[fsn3786-bib-0034] Odunmbaku, L. A. , Sobowale, S. S. , Adenekan, M. K. , Oloyede, T. , Adebiyi, J. A. , & Adebo, O. A. (2018). Influence of steeping duration, drying temperature, and duration on the chemical composition of sorghum starch. Food Science and Nutrition, 6, 348–355. 10.1002/fsn3.562 29564102 PMC5849913

[fsn3786-bib-0035] Oke, M. O. , Awonorin, S. O. , Sanni, L. O. , Asiedu, R. , & Aiyedun, P. O. (2013). Effect of extrusion variables on extrudates properties of water yam flour—A response surface analysis. Journal of Food Processing and Preservation, 37, 456–473. 10.1111/j.1745-4549.2011.00661.x

[fsn3786-bib-0036] Olatidoye, O. P. , & Sobowale, S. S. (2011). Effect of full‐fat soy‐bean flour on the nutritional, physicochemical properties and acceptability of cassava flour. Electronic Journal of Environmental, Agricultural and Food Chemistry, 10, 1994–1999.

[fsn3786-bib-0037] Oluwole, O. B. (2008). Effect of thermo extrusion cooking on physicochemical, textural and sensory qualities of yam (Dioscorea rotundata) and bambara groundnut (Voandzeia subterranean L. Thou) blends. Unpublished PhD thesis, UNAAB, Abeokuta, Nigeria, 117–213.

[fsn3786-bib-0038] Petitot, M. , Boyer, L. , Minier, C. , & Micard, V. (2010). Fortification of pasta with split pea and faba bean flours: Pasta processing and quality evaluation. Food Research International, 43, 634–641. 10.1016/j.foodres.2009.07.020

[fsn3786-bib-0039] Rapusas, R. S. , & Driscoll, R. H. (1995). Thermophysical properties of fresh and dried white onion slices. Journal of Food Engineering, 24, 149–164. 10.1016/0260-8774(94)P2640-Q

[fsn3786-bib-0040] Rosentrater, K. A. , Muthukumarappan, K. , & Kannadhason, S. (2009). Effects of ingredients and extrusion parameters on aqua feeds containing DDGS and tapioca starch. Journal of Aquaculture and Feed Science Nutrition, 1, 6–21.

[fsn3786-bib-0041] Sanni, L. O. , Bamgbose, C. A. , & Sanni, S. A. (2004). Production of cassava instant noodles. Journal of Food Technology, 2, 83–89.

[fsn3786-bib-0042] Singh, R. P. , & Heldman, D. R. (1993). Introduction to food engineering. London, UK: Academic Press, Inc..

[fsn3786-bib-0043] Singh, S. , Raina, C. S. , Bawas, A. S. , & Saxena, D. C. (2005). Effects of heat–moisture treatment and acid modification on rheological, textural and differential scanning calorimetry characteristics of sweet potato starch. Journal of Food Science, 70, 373–378.

[fsn3786-bib-0044] Sobowale, S. S. , Adebiyi, J. A. , & Adebo, O. A. (2017). Optimization of blanching and frying conditions of deep‐fat fried *bonga* fish (*Ethmalosa fimbriata*). Journal of Food Process Engineering, 40(5), e12551 10.1111/jfpe.12551

[fsn3786-bib-0045] Sobowale, S. S. , Awonorin, S. O. , Shittu, T. A. , & Ajisegiri, E. S. A. (2014). Artificial neural network (ANN) of simultaneous heat and mass transfer model during reconstitution of gari granules into thick paste. International Journal of Chemical Engineering and Applications, 5, 462–467.

[fsn3786-bib-0046] Sobowale, S. S. , Awonorin, S. O. , Shittu, T. A. , Ajisegiri, E. S. , Adebo, O. A. , & Olatidoye, O. P. (2017). Modeling of the garification process of fermented cassava mash. Journal of Bioprocessing and Biotechniques, 7, 311.

[fsn3786-bib-0047] Sobowale, S. S. , Ayodeji, O. A. , & Adebiyi, J. A. (2017). Development of a twin screw extruder. Agricultural Engineering International: CIGR Journal, 19, 181–186.

[fsn3786-bib-0048] Sobowale, S. S. , Bamgbose, A. , & Adeboye, A. S. (2016). Effect of extrusion variables on the extrudate properties of wheat‐plantain noodle. Journal of Food Processing and Technology, 7, 547.

[fsn3786-bib-0049] Sobukola, O. P. , Babajide, J. M. , & Ogunsade, O. (2013). Effect of brewers spent grain addition and extrusion parameters on some properties of extruded yam starch‐based pasta. Journal of Food Processing and Preservation, 37, 734–743. 10.1111/j.1745-4549.2012.00711.x

[fsn3786-bib-0050] Sumathy, A. , Ushakumari, S. , & Malleshi, N. (2007). Physico‐chemical characteristics, nutritional quality and shelf‐life of pearl millet based extrusion cooked supplementary foods. International Journal of Food Science and Nutrition, 58, 350–362. 10.1080/09637480701252187 17558727

[fsn3786-bib-0051] Tong, C. H. , Sheen, S. , Shah, K. K. , Huang, V. T. , & Lund, D. B. (1993). Reference materials for calibrating probes used for measuring thermal conductivity of frozen foods. Journal of Food Science, 58, 186–192. 10.1111/j.1365-2621.1993.tb03240.x

[fsn3786-bib-0052] Udachan, I. S. , Sahoo, A. K. , & Hend, G. M. (2012). Extraction and characterization of sorghum starch. International Food Research Journal, 19, 315–319.

[fsn3786-bib-0053] Yam, K. L. , & Papadakis, S. E. (2004). A simple digital imaging method for measuring and analyzing color of food surfaces. Journal of Food Engineering, 61, 137–144. 10.1016/S0260-8774(03)00195-X

[fsn3786-bib-0054] Yusuf, M. , Filli, K. B. , Umar, I. , & Halilu, M. (2017). Effect of extrusion variables on physical properties and acceptability of *Dakuwa* produced from blends of sorghum (*Sorghum bicolour l*), groundnut (*Arachis hypogeal l*) and tigernut (*Cyperus esculentus*). African Journal of Food Science and Technology, 8, 138–149.

